# Intensive Care Nurses’ Experience of Caring in Greece; A Qualitative Study

**DOI:** 10.3390/healthcare11020164

**Published:** 2023-01-05

**Authors:** Stelios Parissopoulos, Fiona Timmins, Meropi Mpouzika, Marianna Mantzorou, Theodore Kapadochos, Eleni Papagaroufali

**Affiliations:** 1 Department of Nursing, School of Health and Care Sciences, University of West Attica, 12243 Athens, Greece; 2 School of Nursing, Midwifery & Health Systems, University College Dublin, Belfield, D04 V1W8 Dublin, Ireland; 3 Department of Nursing, School of Health Sciences, Cyprus University of Technology, Limassol 3036, Cyprus; 4 Department of Social Anthropology, Panteion University of Social and Political Sciences, 17671 Athens, Greece

**Keywords:** caring, empathy, technology, negotiation, ICU nurse, thematic analysis, qualitative

## Abstract

Background: Whilst nurses and critical care services have been at the forefront of the COVID-19 pandemic, it has become more apparent that intensive care nurses are presented with challenging ethical and clinical decisions and are required to care for individuals with critical illnesses under high-pressure conditions. This is not a new phenomenon. The aim of this study, which was conducted before the outbreak of COVID-19, was to explore the experience of caring through the narratives of intensive care nurses in Greece. Methods: A qualitative study was conducted through in-depth, semi-structured interviews with nineteen ICU nurses in Athens. Transcripts were subjected to Braun and Clarke’s thematic analysis and organised with Atlas.ti v8 QDA software. Results: The intensive care nurses’ experience of caring in Greece encompassed four themes: (A) being “*proximal*”, “*co-present*” and caring with empathy, (B) being “*responsible”* for your patient and *negotiating* with the doctors, (C) technology and “*fighting with all you’ve got*”, and (D) “*not being kept informed*” and disappointment. Conclusions: The narratives of this study highlight that ICU nurses in Greece provide patient-centred and compassionate care. Nurse leaders should develop appropriate healthcare policies so as to ensure the adequate provision of staff, specialist education, and support to nurses working in critical care. Failure to address these issues may lead to poor quality of care and negative patient outcomes.

## 1. Introduction

The intensive care unit (ICU) is a technologically advanced microcosm that is physically separated from the rest of the hospital via physical and disciplinary barriers. The ICU presents extra challenges to the ICU nurses, who are expected to not only care for their patients and attend to their physical needs but also care for their psychological and spiritual needs [1,2,3,4]. In the recent COVID-19 pandemic, nurses and critical care services were at the center of the media’s attention, and it became more apparent to everyone, including peer professionals and lay people, that ICU nurses are continually presented with ethical dilemmas and challenging clinical decisions, and are required to care for individuals with critical illness under extremely high-pressure conditions.

Although caring is a concept that has become synonymous with “nursing care” and “plans of care” [5], it still remains a fuzzy and complex term that has attracted various definitions and conceptualizations [6,7,8]. According to Leininger [9], who was one of the first theorists to define caring, caring is a term that describes “those actions and activities directed toward assisting, supporting, or enabling another individual or group with evident or anticipated needs to ameliorate or improve a human condition or lifeway” (p.4). More recently, Tang et al. [10] defined caring as participating in reciprocal interactions, embracing the essence of caring, evoking instances of caring, and embodying caring in practice in the contemporary healthcare system. Brilowski and Wendler [11] identified five key aspects under the caring concept, namely “relationship”, “action”, “attitude”, “acceptance”, and “variability”. Whilst this was tied to the nursing practice, it could also be applicable to other healthcare practitioners. Sebrant and Jong [12] performed a meta-synthesis on the concept of caring and identified four themes: “to be”, “to want”, “to be able to”, and “to do”. Caring was understood by nurses as a movement and balance between these four dimensions, while attempting to actualize what they regarded to be a requirement for good care. It goes without saying that the major role of a nurse is to care for patients; therefore, caring for patients revolves around meeting the particular requirements of patients, and the ultimate goal of nursing is to offer high-quality care [13]. However, the experience of caring is experienced differently by nurses.

Caring for critically ill patients in the ICU requires special considerations. The ICU is a high-pressure setting because of the complexities of the work. ICU settings facilitate the delivery of care to patients who are in the midst of a life-threatening physiological crisis [14]. In his or her job, the nurse serves as a key hub for all activities involving the patient [6]. It is important to take into consideration that caring for patients in an ICU can take many forms [6]. It is worth noting that there have been quite a few qualitative studies into critical care, but they have mostly focused on the experience of certain aspects of ICU nursing, such as managing non-invasive ventilation patients [15], nurses’ perceptions of patients’ thirst in ICU [16], nurses’ perceptions of futile care and caring behaviors [13], nurses’ experiences of caring for dying patients [17], and others [18,19,20,21,22]. A number of studies during the COVID-19 pandemic specifically explored the extra physical and psychological challenges and strains that nurses had been facing [2,23,24,25,26,27], for example, the experiences of cardiovascular nurses working in a COVID-19 ICU [28] and the spiritual well-being of nurses [29]. However, only a small number of qualitative studies have explored the experience of caring as a whole in the context of the ICU [30,31,32,33]. To the best of our knowledge, when this study was conducted, there was no other study that specifically looked at the Greek ICU nurses’ experience of caring as a whole.

In the past, the clinical “landscape” of the ICU has offered a unique opportunity to researchers for inductively exploring nurses’ views and practices that may shed light on how they “navigate” patients through their trajectory of critical illness [34]. More specifically, those studies looked at how nurses *produce* ICU care and reach clinical decisions in moments of access or exclusion at the hospital [35,36], at how they regulate space and time in order to uphold good care and “produce” patient centered care [37], and at how they exercise power in order to secure “medical orders” and care modalities that are appropriate for their patients [35,38,39].

The aim of this study was to explore the experience of caring through ICU nurses’ narratives in Greece. Each interview started with an opening question on what it meant to them to be an ICU nurse and how they experienced what they did. Participants were encouraged to use their own words and share thoughts, feelings, memories, and descriptions. In this study, caring is approached as providing “nursing care” in the context of the ICU. The main researcher (first author), a UK-trained ICU nurse and anthropologist with a Greek background, felt it was important to give the chance to ICU nurses in Greece to share in their own words their experience of caring, as previous studies had suggested that the majority of ICU nurses in Greece were experiencing dissatisfaction, reduced professional autonomy, and reduced participation in their clinical decision-making [1,40,41,42,43].

## 2. Materials and Methods

### 2.1. Study Design

This is a qualitative study that is part of a larger anthropological PhD study conducted in Greece that looked at nursing practice, clinical decision-making, and power relations in the ICU [44]. A qualitative approach was chosen as it is suitable for examining concepts such as “caring”, and it is frequently utilized to highlight the significance of caring from the viewpoints of nurses [45]. Kaba et al. [46] assert that the aim of qualitative research is to explore and achieve a comprehensive knowledge of participants’ perspectives, experiences, and attitudes toward a phenomenon of interest. The goal of qualitative researchers is to get a richer, more comprehensive knowledge of the subject that they are studying.

The main researcher looked at ICU nurses’ experiences of what they did, thought, and felt during a two-year period at a hospital in Athens. This study was conducted in line with critical medical anthropology [47,48,49,50] and phenomenology [51,52,53,54,55,56]. According to critical medical anthropology, which draws largely from the seminal work of Foucault [57], nurses are individuals who co-exist with other professionals in a space (hospital) and they are subjected to a network of powers unfolding through “technologies” of discipline [47,50,57,58]. Therefore, the study adopted an ethnographic approach through fieldwork in order to uncover the discourses, cultures, and practices that transcended an ICU in Athens [59,60]. In doing this, the main researcher conducted in-depth interviews in addition to ad hoc ethnographic interviews with nineteen participants in order to invoke narratives and approach their experiences and representations surrounding their nursing work. This was the phenomenological element of the study. Phenomenology complements ethnographic research in anthropology, by looking at the individuals’ emic perspective and intersubjective experiences of their own world and identity(-ies). This paper reports on particular findings from the analysis of the interviews with nineteen ICU nurses.

### 2.2. Setting and Participants

Nineteen (*n* = 19) participants were selected through a purposive and snowball sampling approach. Eleven of them were ICU nurses who worked at an 18-bed general ICU in a public teaching hospital in Athens, and eight of them were added to the sample as they had been identified as experts in ICU nursing. The “expert” nurses (ICU nurses/managers, nurse academics/researchers in critical care) were recognized as such by other peers and nurses in critical care, due to their achievements and reputations in clinical, academic, and research fields. Experts in ICU nursing were included in the sample in order to ensure maximum variation in the data collection. All of the participants, apart from one novice nurse, had >5 years of clinical experience in ICU (Table 1). In qualitative research, certain “informants” are selected because they have firsthand knowledge of the issue being investigated [46]. Purposive sampling is the term used to describe this type of sampling [61], which can produce rich data. The primary goal of sampling in qualitative research is to find what Patton [62] refers to as information-rich instances, which are “presented for in-depth examination” and from which “one may learn a lot about topics of vital relevance for the purpose of research” (p. 230).

### 2.3. Data Collection

The semi-structured interviews explored the participants’ feelings, thoughts, and reflections on being an “ICU nurse”, with a particular focus on their experience of caring for critically ill patients. The researcher had prepared exploratory, open-ended questions for the interviews in order to elicit narratives and rich information from the participants (Table 2). Additionally, probing and clarification questions were asked during each interview depending on the participant’s responses. Each participant was approached face-to-face to give an interview. The main researcher’s previous clinical background in critical care helped establish, from the very beginning of the study, a sense of understanding and a sense of “being one of them” with the participant nurses in the study. All of those who were approached agreed to participate. In some cases, the privacy and “stillness” of time during the interview session offered them a chance to pause, reflect, offload emotions, and share their own intersubjective—but very real to them—embodied experiences of caring. In social anthropology, embodiment is a way of describing enlivened bodily experiences. In other words, the body is more than an object [51,52,55], it “is also a locus from which our experience of the world is arrayed, it is a living entity by which, and through which, we actively experience the world” [63] (p. 89).

The participants selected the place and time of the interviews (home, hospital, work office, or university). Privacy was ensured, and the places were quiet. Each interview lasted approximately one hour, was recorded, and was transcribed verbatim by the main researcher. Finally, interviews were completed once data saturation was reached.

### 2.4. Ethical Approval

Participants were informed in advance of the purpose and design of the study and signed an informed consent form. Participation was voluntary, and each informant had the right to withdraw at any time. Their willingness to participate in the study was reconfirmed verbally before beginning the interviews. Ethics approval was obtained by the Ethics and Research Committee at the hospital. Particular care was given to protect the participants’ identities, by using pseudonyms and sensitive patient information according to the principles of confidentiality.

### 2.5. Data Analysis

Interviews were analysed inductively and coded line by line. Coding gradually reached higher levels of abstraction, and the interviews were grouped together to form code groups (categories). The transcripts were subjected to Braun and Clarke’s thematic analysis of qualitative data [64]. Braun and Clarke’s thematic analysis constitutes an independent qualitative approach for an analysis identifying, analysing, and reporting patterns (themes), but is also characterized by flexibility and compatibility to phenomenological research, which was important in this study [65,66]. The stages of thematic analysis are divided into: (a) familiarity with the data, (b) generation of initial codes, (c) search for codes of higher abstract level and generation of code groups, (d) review of codes, (e) definition and naming of themes, and (f) writing on findings [64,67]. As Braun and Clarke point out in their methodological paper on thematic analysis [64], “A theme captures something important about the data in relation to the research question, and represents some level of patterned response or meaning within the data set... the ‘keyness’ of a theme is not necessarily dependent on quantifiable measures—but in terms of whether it captures something important in relation to the overall research question.” (p. 10). Table 3 presents an example of coding and analysis of an interview transcript. This process of grouping, categorizing, and theorizing the data is very common in qualitative research and helps the researcher “make sense” of the data and reach meaningful interpretations and descriptions. In this study, interviews were analysed simultaneously with the emerging interpretation of the data. The supervisory team of the PhD study discussed, checked, revised, and adjusted “emerging” themes and patterns, on an ongoing basis.

### 2.6. Rigor of the Study

Trustworthiness was ensured by satisfying the principles of credibility, confirmability, dependability, and transferability [66,68,69]. Credibility was established through the triangulation of data methods and sources. Firstly, ethnographic fieldnotes were kept during participant observation. The ethnographic part of the study helped the main researcher identify the key informants for interviewing. Secondly, the sample included experts in critical care. Transferability was met by adopting purposive sampling. Confirmability and dependability were satisfied by keeping a research diary, recording methodological decisions for the purposes of audit trail, and member checking. The findings, even at preliminary stage, were discussed with the participants for cross-checking and clarification. The data analysis and revision of the themes carried on well into the write up process of the thesis. The audit trail included reflective writing and ongoing supervisory support from the PhD supervisors. The standards for reporting qualitative research (SRQR criteria) guided the preparation of this paper [70].

## 3. Findings

The ICU nurses’ experience of caring in this study encompassed the following four themes: (a) being “*proximal*”, “*co-present*”, and “caring with empathy”, (b) being “*responsible*” for your patient and *negotiating* with the doctors”, (c) technology and “*fighting* with all you’ve got”, and (d) “*not being kept informed*” and disappointment (Table 4).

### 3.1. Theme A: Being “Proximal”, “Co-Present”, and Caring with Empathy

**Figure 1:**
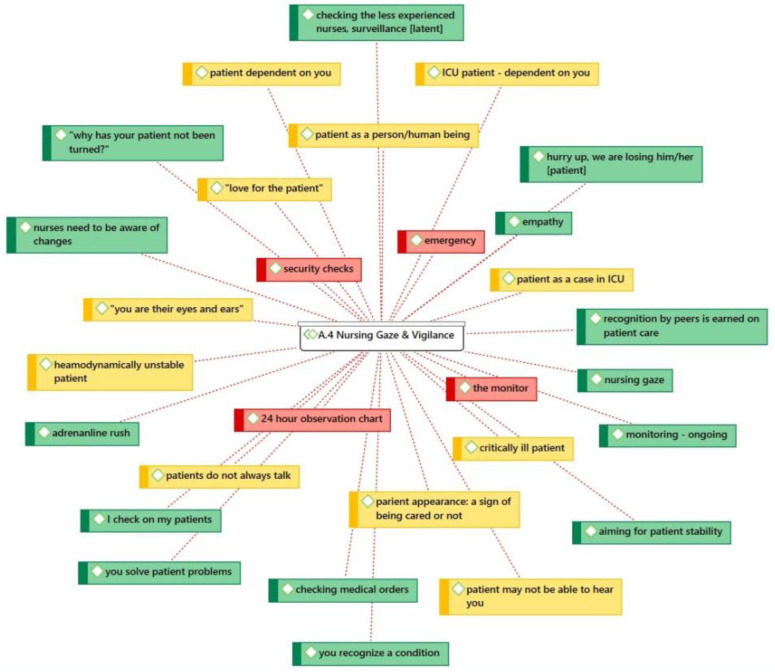
Nursing gaze and vigilance.

The participants perceive themselves as individuals who are “proximal” to their patients and continuously “co-present”. This type of constant co-presence and closeness describes a nurse-patient relationship that is not just metaphorical but very much physical and embodied. A nurse is always present and available in the patient’s area and is vigilant of any physiological changes or alarms going off. This type of nurse-patient relationship can also be described as an empathetic one. “Nursing Gaze and Vigilance” was an important finding (code group 4) and is depicted with its associated codes in Figure 1.

ICU nurse Akrivi is “co-present” and “proximal”, to her patient, both metaphorically and literally. She constantly has her patient in her mind, “she cares for him”, and does what is necessary to keep him alive. Her vigilant eye makes sure that all their bodily functions are monitored and recorded, and that no major functions, such as airway patency, breathing, and blood pressure, are failing the patient. At the same time, she feels the patient’s pain and suffering, she speaks to him, supports him, touches him gently with her hands, and reassures him. The participants expressed empathy for their patients’ needs and problems, especially when verbal communication with the patient was not possible, due to the threatening critical illness or sedation.

*Akrivi, interview 2: “Sometimes I think of my patients very intensely, I think about their situation, yes, and I think about it very intensely… it affects me very badly […] I may be sitting and looking at them and I think ‘this man is in bed, motionless, I wonder if he is a little awake, if he is in pain’”.*

ICU nurse Agne remembers how intensely she had experienced a difficult case, the “Christmas miracle” patient. A multi-trauma 32-year-old woman, mother of a two-year-old girl, had been transferred years ago to the ICU on a Christmas Eve. She had finished work and was waiting to be picked up when two cars collided in front of her. One of the cars hit her and she lost her leg. She was admitted post-op to ICU with one leg amputated from the thigh down, an unstable pelvic fracture and she was in very critical condition. Agne remembers that seven hours had passed since the patient’s admission, and that she herself had been stubborn and took it for granted that the patient would do well. She was so involved in her care that she could not accept that her patient would not make it. While the young woman was losing blood non-stop and was being transfused with many units of blood and plasma, Agne and the doctor kept “working on her” with all their physical, cognitive, and emotional resources.

*Agne [expert nurse] interview 9: “I remember transfusing her non-stop, I am saying this to you and I am shivering… I remember both of us (Agne and the doctor) holding the blood units and applying pressure to deliver the blood as fast as possible. I remember a colleague coming on duty and saying ‘why give so much blood? since she will not make it.’ And I didn’t like the sound of it, I remember thinking ‘what is she saying? she will survive!* *’”*

Agne was so involved and connected to her patient, that she strongly believed there was a real chance of her survival. The next morning, she was still alive, still critical but still there, and she was discharged from the unit a few weeks later. When she thinks of her, seven years later, she thinks of her as a mom at home, with one leg, but having a life. Her belief that this was a case that had a chance of survival kept her “fighting” on the patient’s behalf.

### 3.2. Theme B: Being “Responsible” for Your Patient and Negotiating with the Doctors

Whilst nurses care for their patients, they engage in creating space for negotiation with the medical team in order to achieve the best possible interventions for their patients. Like most of the participants, one of the reasons nurse Artemis considers herself responsible for the patients assigned to her or under her care is that she is accountable for them at the end of the shift. As in other studies, her sense of responsibility and accountability for her patients is a profound element of her job.

*Artemis, interview 1: “As I have to account for the patients assigned to me and I sign off their charts and documentation, I consider myself responsible for these patients. However, if I pick up something wrong with the patients of a colleague, even if he is a senior/experienced one, and I know I just said ‘his patients’, I will still intervene because the patient comes first! [laughs]”*

All participant nurses felt enormously responsible for the patients who were under their care. Interviews revealed the existence of an “intense relationship” with their patient. All participants often referred to their patients as “theirs”, as if they “owned” them. However, according to Agne, this sense of responsibility was experienced as an extra burden for them, as their workload was heavy, and they also had to keep an eye on the patients of the less experienced colleagues. Therefore, the participants also “supervised” and exercised some kind of control over their colleagues’ work and the patients that had been assigned to them.

For some of the nurses, the sense of responsibility for their patients is so great and heavy that it is embodied as a significant emotional burden. The participants in the current study made themselves available and mobilized their body, their senses, and their “nursing gaze” for the best interest of the patients under their care. All participants utilized basic and advanced clinical skills, and each time they cared for their patient, they produced nursing care that was “culturally” suitable to the lifeworld of ICU.

Although they carefully shielded themselves behind protective personal equipment (PPE) with face masks, gowns, and gloves, every “touch” and act of “care” was carried out with a sense of responsibility, accountability, and intention to heal, such that in a latent fashion it ensured that the patient under their care was not alone in this precarious physical state between life and death. Erasmia tries to make her presence known to her patients, even through a gentle touch.

*Erasmia, interview 10: “yes, I am consciously there… this is why you see me touching my patient, you see the ‘touch’. I may be passing by my patient and even though I am not verbally communicating to him, because he is sedated, I intentionally touch him. It is my way of telling my patients ‘I know and I care for you, I am here for you’”.*

Nurses’ sense of responsibility for their patients gave rise to the creation of a space for negotiation between nurses and doctors. The analysis of the data in the present study confirms that the daily life in the hospital and in the ICU seems to be organized around a series of negotiations between nurses and doctors. Artemis is in constant negotiations with doctors, especially now that she is experienced and she feels a greater sense of responsibility. Artemis negotiates for almost everything: for the patient’s analgesia, whether she will “wake up” the patient or not, or when a new “line” will be inserted in one of her patients. Scheduling invasive procedures and computerized tomography scans by doctors without warning or at least telling nurses was a constant source of tension between nurses and doctors, as the nurses had three patients under their care, and inappropriate scheduling of such interventions meant that they could not keep up with their ongoing “nursing” jobs. While Artemis could not do the same in the first years of her career, she has reached a point where she “controls” the doctors and can have a say in the scheduling of such interventions.

*Artemis, interview 1: “after ten years of experience in critical care, I have the sort of relationship with the doctors where I can tell them what needs to be done, I can control them. It is not like 5 years ago where I would not question their orders. Now I am able to question their decisions and negotiate a timeframe which is possible for me to manage”.*

### 3.3. Theme C: Technology and “Fighting with All You’ve Got”

Technology in ICU has a prominent place in the provision of care, as it helps nurses and doctors in “invading” and looking at the physiological functions of the body, in the administration of therapeutic interventions, and in reaching clinical decisions based on up-to-date data. The primary concern of the participants was to provide lifesaving and supportive care, to stabilize the patients’ clinical condition, to recognize clinical deterioration, and maintain homeostasis in their patients. According to nurse Elpida, nurses can formulate an opinion on the patient’s severity just by looking at the number of electronic medication pumps and the type of machines attached to the patient (filter). In the *indigenous* language of ICU nurses, the patient is often described as having “full” or “empty” equipment on his bedside.

*Elpida [expert nurse], interview 6: “I do not think we actually acknowledge the vast number of machines we connect to our patient, because we are so used to this. It is shocking for someone who sees this for the first time, but because we are so used to the image of a patient being attached to IV pumps, monitor, ventilator… we do not take much notice”.*

**Figure 2:**
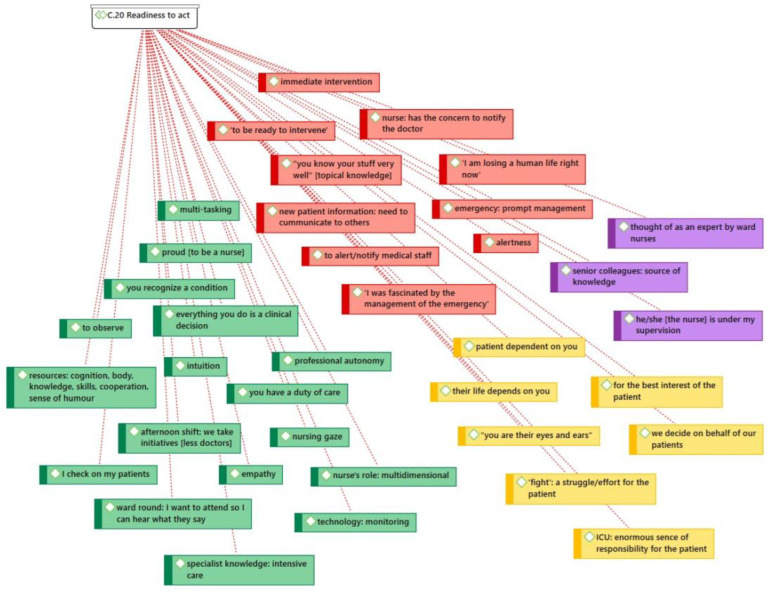
Readiness to act.

Expert ICU nurse Elpida believes that nurses utilize and mobilize “all they’ve got”, such as their knowledge, skills, and humor, in order to achieve patient stability and body homeostasis. The participants also placed particular emphasis on their readiness to act (code group 20) in the event of patient deterioration (Figure 2).

Nurse Elpida describes the experience of caring as a “battle” where the nurses strive with all their “resources” to achieve the best outcome for the patient. At the same time, nurses work closely with the other professionals, they negotiate, they employ their sense of humor, and if needed, they assume a stricter and “authoritarian” approach.

*Elpida, interview 6: “at the time of the cardiac arrest [laughs]… at that time you are doing things that you thought you could not do and you are using whatever skills you have in this ‘battle’, that is cooperation with others… humor… every time you use different resources”.*

Quite often they had to manage faulty and malfunctioning equipment, for example, intravenous cannulas that broke easily, giving sets that “refused” to work, even at a time when their patient was bleeding and “dropped the pressure”. At the time of the study, the participants were extremely resourceful in thinking of patients and improvised ways of using this material, as they had to come up with innovative ways in order to provide the best possible treatment. Nurses joked about this, and they described Greek nurses as “cockroaches who survive and manage through tough situations”.

### 3.4. Theme D: “Not Being Kept Informed” and Disappointment

The nurses in this study were sometimes overwhelmed with disappointment and frustration as the medical staff didn’t keep them informed of any changes in treatment or patient background. So, even though they were constantly “present” and “close” to their patients and knew the smallest details of their conditions, they often felt that they missed the big picture, and were left out of important clinical decisions. In addition to this, each time the nurses failed to “convince” the doctor that there was something wrong with their patient, they were overwhelmed with frustration, sadness, and anxiety. Therefore, even though some of the participants experienced immense job satisfaction, a strong “sense of helping” the others, a “love for” and, to some degree, an “addiction” for the ICU specialty, some others were very disappointed, frustrated, and felt devalued, and they considered some of the tasks delegated to them of minor importance. Their main complaint was their inability to attend ward rounds due to extreme workload, as each nurse was delegated three patients. Galenos, an experienced nurse, feels like an “intruder” on the few times he has tried to attend the doctors’ ward round. He sits somewhere on the edge, listens to them quietly, and when he finds the opportunity, he may ask questions. However, often “there is no time” for Galenos to do so, and he continues with his own work and patient care.

*Galenos, interview 15: “No there is no time… we cannot go [to the ward round]… I cannot afford to sit around for 40–60′ for my three patients, right? …they do not have a set time for the round. As you cannot arrange your work, you cannot be available for ward round without enough warning. If I knew that the round is scheduled for ten o’clock, I would had arranged to finish my jobs by ten o’clock and then join them. But now, you cannot do it”.*

In general terms, the nurses in this study disclosed that doctors would not inform them of the details of the patient’s previous medical history, investigations, and progress. This made them feel undervalued and unable to participate in both the ward rounds and multidisciplinary clinical decisions. The latter led them to experience a state of nurse invisibility and “absence” from the clinical decisions of the ICU team. Nurse Galene does not hesitate to admit that she, as well as her colleagues, rarely participated in the ward rounds, although she knew it was important for her patients. Galene realized that medical staff would not consult the observation chart to the full extent, and she would like to be part of the ward round and bring areas of concern to the attention of the doctors. Herein lies the frustration of Galene, who, although she sees herself as the “eyes” and “ears” of doctors and as a useful source of information, doctors do not consider her “important” enough to be present at the ward round. Heavy workload (ration 1:3) and fatigue further discourage her from participating in the rounds.

*Galene, interview 4: “The workload… because most of the times, when my work is too much and I am thinking of the many pending tasks I don’t want to pass on to my colleagues, I don’t have the ability to go to the round...”*

According to the participants, the medical team often carried out their ward rounds on their own at ad hoc times and often in their office and the corridor, away from the nurses and the patients. Nurse Akrivi is tired and frustrated that she is distrusted by some of the doctors that she has worked with for so many years. According to her, her fatigue and efforts to provide high-quality care for the three patients under her care went unnoticed. I asked her if there were problems at work.

*Akrivi, interview 2: “[-] [long pause] there are problems. I…. there are too many… that disappoint me too much. The disbelief and [….] many times the irony of the doctors towards us... [….] it happens a lot… [silence]… I see this, it frustrates me very much and I am very tired in the Unit [….] especially when you know that you do right all the basics and you follow the procedures even though you do not have the resources”*

## 4. Discussion

**Figure 3:**
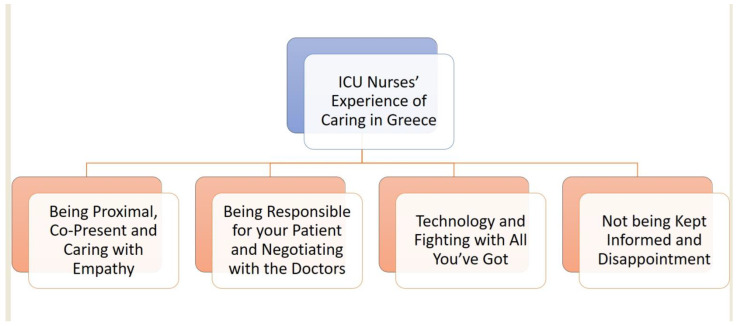
The main themes of the study.

The findings of this study capture the experience of caring among ICU nurses in Greece through the analysis of their narratives (Figure 3).

The first theme [A] suggests that the participant nurses in this study ensure that they are *close* (proximal) to their patients, are *co-present,* and demonstrate *empathy* while caring for them. In relation to proximity, in Lundin Gurné’s study [71], proximity came up when nurses were described as working more closely with patients than other professions. The participants in that study emphasized that nursing practice was about striving to be in close proximity to the patient. This proximity was not easy to achieve, as nurses were often instructed to perform tasks not traditionally performed by nurses or not clearly perceived as nursing tasks. Nevertheless, they strived to be close to their patients, even though they had less time to care for individual patients due to cuts in healthcare funding [71]. It is worth noting that proximity to the patient enhances the nurses’ bargaining role in clinical decision-making [72].

The critically ill patient requires the presence of a trained ICU nurse all the time in order to monitor and detect any rapid changes or deterioration. Being co-present also meant being available for the patient [6,71]. In Lundin Gurné’s et al. study [71], the nursing practice was described as being constantly available to patients and their relatives as well as colleagues while also focusing on patients’ needs. This was also an important finding in the current study. According to Al-Shamaly [6], the imperative necessity for patient safety demands nurses to be “present with” the patients, “conscious of the non-verbal care demands,”, and “sensitive” to the subtle shifting health states of patients. All of these elements are recognized as essential components of patient care in critical care [6]. In the ICU, nurses are concerned with ensuring patient safety for all patients, but especially for the conscious patients who are receiving mechanical breathing [73,74]. When Beeby [30] described the experience of caring among ICU nurses in a UK hospital, the participants experienced caring as “being involved in care” and “sustaining support” but also as “having frustrations”. In terms of being involved, they meant that it was important for them to “be close” to the patient, to “be there”, and to “do things for the patient”. “Sustaining” referred to the nurse’s role in providing support to the patient. The nurse provided a foundation of care that allowed the patient to recover by maintaining a stable environment.

According to Rogers, empathy is the ability to momentarily *step into* and *inhabit* another person’s life by emotionally and physically *occupying* their personal space with the intention to help [75]. In nursing, caring with empathy also means “I do something for it”, “I am available to the patient, I develop a therapeutic relationship and I respond to his needs”. Empathy is also required for developing a therapeutic relationship and responding to the needs of others [22,76]. This type of nurse involvement was also found in the phenomenological study on ICU nurses by Vouzavali et al. [20], where the patient-nurse relationship in critical care was described as a “shared world”, in other words, as a strong bond/synapse between “neighboring cells” that form a “syncytium”. In the study of Stavropoulou et al. [3] on ICU nurses in Greece, care with empathy was described as sustaining communication, providing support, and a necessary condition for maintaining the patient’s body homeostasis. ICU patients and their families are well-known for feeling powerless, vulnerable, anxious, and stressed [77]. Empathy in critical care encompasses acting in the best interest of the patient and results in caring with compassion [78].

The second theme of the study [B] demonstrated a profound sense of *responsibility* on the part of the participant nurses, which was linked to *negotiation* with doctors. Nurses negotiate with doctors in order to influence or secure the best possible clinical decision for their patient. Creating space for negotiation was a priority. In the spirit of this, nurses remained vigilant and often took on responsibilities that traditionally belonged to doctors [79] to promote the safety and homeostasis of “their” patients’ bodies. The nurses claimed that their responsibility for *their* patients played an important role in this. In Beeby’s study [30], caring was also experienced as accepting responsibility for a completely dependent patient and being at the bedside to provide patient care. Caring entailed trying to meet the individual’s needs and, despite the gravity of the condition, acting as the patient’s advocate within the ICU team. Their obligation to their patients informs their clinical decisions, even when they encounter ethical dilemmas in practice [19,80,81]. In another ethnographic study on the cultural context of an ICU in South Africa, Scholtz et al. [33] argued that nurses “adopt” their patients during their stay in the ICU. That is, they feel that they take full responsibility for them. As a result of this, they took any kind of difficulty or bad outcome very personally. Short pointed out a long time ago [82] that nurses see their bodies as tools of work and the sick bodies of their patients as the work area. Caring is experienced with a sense of responsibility for the monitoring and safety of their patients. For example, sharing both observation responsibility between colleagues and their perception of the patient’s clinical condition with other professionals is part of teamwork skills [18]. Responsibility also meant that nurses had to negotiate with doctors on care interventions or drug modification and titration. During negotiation, nurses use communication strategies that encounter issues of concern and problems that cannot be regulated by written rules and institute policies [36]. In Schluter’s et al. study [36], competent nurses were considered those who not only were close to their patients but those who effectively negotiated their ideas and opinions with the medical staff.

This study suggests that the participant nurses negotiate with doctors in order to influence or secure the best possible clinical decision for their patient, which also supports the findings of the study by House and Havens [83], where the participants were found to engage in communication and negotiation techniques in order to overcome difficult interdisciplinary collaborations. In other words, it offers support to Coughlin’s statement [84], which describes nurses’ work as navigating the patient through his/her illness trajectory in the ICU. This is a rather important finding in the Greek context, where the healthcare system has been described as medically centered with no recognized ICU course for nurses in place at the time of the study. Nevertheless, participants described how they engaged in negotiation techniques and tried to overcome difficult interdisciplinary collaborations. A study by Papathanassoglou et al. [42] has revealed moderate autonomy in technical tasks and low decisional autonomy among Hellenic ICU nurses. Overall, the findings offer support to other studies and suggest the existence of what Hasse describes as a person-centered approach [85].

The third theme [C] highlights that technology and resources, such as skills and specialist knowledge, were perceived by the participants as essential tools in achieving the patient’s body homeostasis. High-quality care for critically ill patients not only requires humanistic methods of care but also a substantial dependence on the most current technology and knowledge [7,86]. It is a fact that ICU nurses cannot produce nursing care without the use of advanced technology. Price looked at the experience of caring in ICU in relation to technology in the UK [32]. She argued that the technology in the ICU cannot be separated from care, as the ability of nurses to handle technology is considered a profound skill and a prerequisite for care. Technology has changed the way we perceive and look at the human body, resulting in the creation of a new “medicalized” body [87]. It is interesting to note that studies have shown that technology has led to wider responsibilities for nurses in decision-making [88,89]. In other words, ICU nurses were shown to acquire a sense of superiority in their role due to the special knowledge they possessed and their unparalleled familiarity with the technology [32,79]. This enhanced their role in clinical decision-making as they were perceived as technical experts who are called upon to manage patients suffering from severe and threatening disease [88,89]. Since then, nurses have been recognized as an important element in providing a high level of critical care and decision-making [6,90]. Experienced ICU nurses are rather vigilant and look out for minute changes in the patient’s condition. Patient monitoring is a key component of modern critical care, and it accounts for a significant percentage of an ICU nurse’s work [91]. However, a critical care nurse is not only a technologically competent practitioner but a specialist who has the skills to decipher complicated data, offer therapeutic benefit through presence and comfort measures, create an active program of care for seriously ill patients, and assist other members of staff in providing critical care [92]. However, Pattison points out that the recent implementation of “task-teams” for the delivery of basic care during the COVID-19 pandemic may lead to the “reduction of nursing to tasks and the erosion of what it is to be a critical care nurse” [92] (p. 422). This new phenomenon goes against the notion that critical care nurses should “focus on the tasks that require advanced expert skills, expertise and knowledge of best practice in patient care” [93].

Lastly, the fourth theme [D] suggests that the participants were not valued to the degree they wished by the medical staff of the unit. A heavy workload (nurse-patient ratio 1:3) and their inability to attend the ward rounds made them feel undervalued, as they felt they were unable to participate in the ward rounds. The latter led them to experience a state of nurse “invisibility” and “absence” from the clinical decisions of the ICU team. Reflecting on the responses and accounts of the participants, no differences were noted between male and female nurses. For example, male nurses complained in the same way as their female colleagues about not being able to participate in the doctors’ rounds. This might be attributed to both male and female nurses sharing the same values and ethos of care in nursing. Moreover, feelings of disappointment were shared among all the participants, as they recognized they were not participating in the clinical decisions of the ICU team. Clinical decision-making has always been an important aspect of ICU nursing [90]. Whether nurses contribute or not to the clinical decisions of the ICU team is a long-standing issue. Notably, there is evidence of nursing invisibility in clinical decision-making [94,95,96,97,98,99]. Studies have shown that nurses’ assessment findings and judgements are either diminished or ignored [94,100]. When this happens, open confrontation, latent tension, and poor team collaboration can be the result [83,101,102]. This is problematic, as disturbed dynamics in the healthcare team and dysfunctional communications are associated with burnout [103] and low job satisfaction [4,104]. On the other hand, successful patient care and improved outcomes are associated with healthy communication amongst the members of the team [105].

This theme addresses the emotions of the participants. Emotions are important in qualitative research as they may shed light on the culture of a hospital setting. For example, a recent number of qualitative papers brought forward the extra physical and psychological challenges and strains that nurses experienced during the COVID-19 pandemic [2,23,24,25,26,27]. Nurses around the world were facing dread, anxiety, stress, physical tiredness, and a sense of powerlessness in dealing with their patients’ situations [106]. Moradi et al. [26] studied the difficulties encountered by 17 ICU nurses while caring for COVID-19 patients. ‘Organizational inefficiency in supporting nurses’, ‘physical exhaustion,’, ‘living with uncertainty,’, and ‘psychological load of the disease,’ were experienced by the nurses. Nurses are also likely to suffer from psychological issues such as anxiety, depression, sleeplessness, stress [107,108], and burnout [109]. Chronic occupational stressors such as high patient acuity, high levels of responsibility, working with advanced technology, caring for families in crisis, and being involved in morally unpleasant situations are regular features of nursing work [3]. As a result, compassion, fatigue, and burnout may lead to a decline in empathy as a self-protective response. This could make their work more emotionally taxing as they become more engaged or emotionally involved. These issues may result in a “care burden” [19].

## 5. Limitations and Strengths

This study has some limitations. Data collection took place before the outbreak of COVID-19, and as the clinical landscape in critical care has substantially changed in the last two years, the findings could not be extrapolated to the experiences of nurses working in the era of the COVID-19 pandemic. Nevertheless, as there are very few studies on the whole experience of caring in critical care, the authors feel that the narratives of this pre-COVID-19 study can still contribute meaningfully to the worldwide discussion that focuses on *caring* and the essence of intensive care nursing [92,93]. Caring is a global concept, and this study sheds light on how ICU nurses in Greece care for and “navigate” their patients through their trajectories of critical illness. In retrospect, the inclusion of “expert” nurses in the sample enriched the data with their “expert” perspective and past clinical background and helped contextualize the accounts and narratives of the participants rather than interpret them in isolation. We suggest that further qualitative research should explore the experiences of caring from nurses’ and patients’ perspective, in order to explore what ICU nursing and care might look like after the COVID-19 pandemic.

## 6. Conclusions

This study concludes that nurse proximity, co-presence, caring with empathy, and a sense of responsibility are the core qualities that constitute the experience of caring among a group of ICU nurses in Greece. The study highlighted that the participants provided patient-centred care with empathy, engaged in negotiation with medical staff in order to affect clinical decisions in the best interest of the patient, and were resourceful through the use of technology, skills, and knowledge. However, even though they perceived that they engaged in a wide spectrum of interventions that stretched from monitoring to supporting and treating patients, they expressed feelings of disappointment regarding their exclusion from doctors’ rounds and non-participation in the clinical decisions of the ICU team. As there has been a long-standing discussion on the power relations between the male-dominated medical and female-dominated nursing professions [58], it is suggested that future research also explore issues of gender regarding the division of therapeutic work between the two professions.

Finally, this paper reveals a strong culture of compassionate care among the participant nurses, even though they provided care under very challenging work conditions with a heavy workload. At times of extreme low nurse staffing levels in critical care across the globe and the recent implementation of “alternative staffing models” by assigning nurses without an ICU qualification [92] (p. 421) to care for ventilated COVID-19 patients, it is imperative to explore and continue the discussion on what actually constitutes ICU nursing and how care is experienced by the nurses in various cultural contexts. The authors suggest further studies that explore ICU nurses’ experiences of caring. Such research would not only contribute to the ongoing discussion on the physical and psychosocial dimensions of what constitutes ICU nursing but would also inform and prepare leaders and nurses for future changes in the organization of critical care services and approaches to care.

## 7. Implications for Practice

This study provides valuable insight into the experience of caring through ICU nurses’ narratives in a southern European country. Nurse leaders that aim to support the wellbeing of their nursing workforce should empower and support critical care nurses and allow decision-making freedom so as to encourage autonomous practice. They should encourage empathy, group spirit, mutual respect, trust, and support within the nursing workforce culture. The development of appropriate policies should also consider crucial organizational issues such as workload, resources, and safe staffing levels [110]. The challenges that nurses currently encounter worldwide are begging for the redrafting of social care and health policy. Not only should nurse leaders respond to their concerns, but health policy makers should also ensure the adequate provision of nursing staff, encourage specialist education in critical care, offer adequate resources, and provide in-house support to nurses working in critical care. The failure to address these issues may have an impact on staff retention and the mental health of nursing staff, which may lead to poor quality of care and negative patient outcomes.

## Figures and Tables

**Table 1:** Participant characteristics.

Pseudonym	Age Group	Gender	Professional Capacity and Education	ICU Experience (Years) at the Time of the Interview	Expert Nurses in Critical Care
Artemis [#1]	25–34	F	ICU nurse, Bachelor’s degree	10	
Akrivi (Precious) [#2]	25–34	F	ICU nurse, Bachelor’s degree, Master student	7	
Doukissa [#3]	25–34	F	ICU nurse, Bachelor’s degree	13	
Galini (Calm) [#4]	25–34	F	ICU nurse, Master’s degree	10	
Stefanos [#5]	25–34	M	ICU nurse, Master’s degree	6	
Elpida (Hope) [#6]	35–44	F	Former ICU nurse, PhD, Clinical Education	9	Expert #1
Marcella [#7]	35–44	F	Former ICU Sister, Master’s degree, Phd(c), Public Health	10	Expert #2
Cleopatra [#8]	45–54	F	ICU Sister, Master’s degree, Phd(c)	19	Expert #3
Agne (Pure) [#9]	35–44	F	ICU nurse, Master’s degree	10	Expert #4
Erasmia [#10]	35–44	F	Former ICU nurse, Master’s degree, PhD(c), Clinical Education	14	Expert #5
Gregoris [#11]	35–44	M	ICU nurse, Bachelor’s degree	6	
Monica [#12]	35–44	F	Former ICU Debuty Sister, PhD, Academic	13	Expert #6
Thales [#13]	35–44	M	Former ICU nurse, Master’s degree, PhD(c), Academic	10	Expert #7
Olympia [#14]	35–44	F	ICU Debuty Sister, Bachelor’s degree	13	
Galenos [#15]	35–44	M	ICU nurse, Bachelor’s degree	6	
Sotiris [#16]	45–54	M	ICU Nurse Manager, Bachelor’s degree	22	
Elpiniki [#17]	25–43	F	ICU nurse, Bachelor’s degree	1,5	
Metaxia [#18]	35–44	F	Former ICU nurse, PhD, Researcher, University	7	Expert #8
Elizabeth [#19]	45–54	F	Matron of Critical Care Division, Bachelor’s degree, Master student	20	

**Table 2:** Overview of the questions in the interview guide.

What does it mean to you to be an ICU nurse? Please feel free to use your own words.
If you reflect on your experiences throughout your nursing career, what kind of thoughts could you share with me?
Hοw do you experience what you do? How do you see your role in your unit? Thoughts, feelings, memories, and descriptions.
Could you describe to me a typical shift in the unit? What is it that you do as an ICU nurse?
If I asked you to remember/recollect a particularly difficult/bad shift, what are your thoughts and feelings?
Is there something that bothers you/puts you under stress/presents you with a dilemma in the ICU?
What brings satisfaction to you at work?
Could you please recall a case/patient where you felt you had made a significant contribution?
Clinical decision-making: what kind of decisions do you usually make? Are you having difficulties? Could you mention something that helps/encourages you to reach a decision or participate in a decision? Is there something that makes it harder for you to participate?
Do you think that your professional judgment is considered by the doctors of the unit?
Intensive care unit: what were your first thoughts and impressions of the ICU environment? How did you feel when you started working in critical care? What impressed you, and what scared you?
Would you describe to me your relationship with the doctors of the unit?
Would you describe to me your relationship to the patient?
Let’s say that the patient’s condition is deteriorating. You are worried about your patient. How would you react/intervene? If medical staff do not act or react the way you had hoped (i.e., they don’t take your assessment findings seriously), what do you do?
Have there been times when you thought that the decisions of the treatment team conflicted with your own values/priorities? How do you handle such situations?
How would you describe the ICU environment to someone who is not familiar with it?

**Table 3:** An example that illustrates the stages of thematic analysis with operational definitions.

Interview Transcript ^a^	Initial Codes ^b^	Codes of Higher Abstract Level-Code Groups ^c,d^	Themes ^e^
*Elpida #6: “You give your own fight for your patients, the way I picture it, it is the effort you’re making at that time and usually ‘fighting’ comes to my mind when I think of cardiac arrests, in the cardiology unit we have a lot of arrests, you pick up a lot of tachycardias, a lot of things, a lot of things like that, and when on duty you make use of everything, you use your mind, you use your body, you use the knowledge you had, you use the skills, skills you didn’t know you had [she laughs], I mean, it might have taken you the whole shift trying to unscrew a stopcock [fluid flow control valve] and in the event of a cardiac arrest you unscrew it just like that [laughs]… but in the event of emergency you do things that you thought you couldn’t do and you use whatever skills you have in this battle, that is, you use your abilities for cooperation, you also use humor, and sometimes to say things you normally wouldn’t say. I remember saying to a doctor, “please get going, please get going, do something” [we laugh]. That is because you also need this [humor]. You also have to be authoritarian sometimes, I think what you use is all you’ve got in terms of skills, every time you use different things, but they all do the same thing. So, you work against time, I mean, you try to catch up, that is, you have things on your mind that you want to get done in your shift-maybe not a lifesaving thing, if it is a quiet shift-but still having a lot of things to do, so you have to manage your time”*	*Fighting for your patient* * *The effort* *Fighting* *Emergencies* *Tachycardias and body* *You make use of everything* *Skills* *Mind and body* *Knowledge* *Skills* *Humor* *Emergency-rapid deterioration* *Skills and battle* *Ability to cooperate* *To the doctor: “get going”* *Humor* *Authoritarian* *Skills* *Time management* *Trying to catch up*	11. Responsibility and accountability 12. Communication: nurse-doctor 14. The emergency: body homeostasis 18. Skills, knowledge, and emotions 20. Readiness to act 21. Fighting for your patient	**B. Being responsible for your patient and negotiating with the doctors** **C. Technology and “Fighting with all you’ve got”**

* Red codes → red code groups → theme (C), green codes → green code groups → theme (B). Codes are colored for illustration purposes, as codes may be assigned to more than one code group. The stages of thematic analysis, [64] operational definitions. ^a^ Familiarizing with your data: transcribing data, reading and re-reading the data, noting down initial ideas. ^b^ Generating initial codes: coding interesting features of the data systematically across the entire data set, collating data relevant to each code. ^c^ Searching for codes of higher abstract level and collating all relevant codes into code groups [categories]. ^d^ Reviewing codes and code groups [categories]. ^e^ Searching for themes, reviewing them, naming themes: collating code groups into potential themes, gathering all data relevant to each potential theme. Checking if the themes work in relation to the coded extracts.

**Table 4:** Overview of themes and code categories.

	Themes	Code Groups [Categories]
**ICU Nurses’Experience of Caring in Greece**	A. Being *proximal*, *co-present,* and caring with empathy	The critically ill patient Monitoring and care: ongoing Nurse being fully there Nursing gaze and vigilance Relationship: nurse-patient Proximity Co-presence
	B. Being *responsible* for your patient and *negotiating* with the doctors	8. *My* patient: worrying/stress 9. The “care needs” of the patient 10. Clinical decision-making 11. Responsibility and accountability 12. Communication: nurse-doctor 13. Negotiation 14. The emergency: body homeostasis 15. Observations and the “observation chart”
	C. Technology and *fighting with all you’ve got*	16. Alarms and equipment 17. Nursing expertise 18. Skills, knowledge, emotions 19. Technology 20. Readiness to act 21. Fighting for your patient 22. “We survive like cockroaches do”
	D. *Not being kept informed* and disappointment	23. Lack of information 24. Absence from doctors’ round 25. Clinical decisions: not participating 26. Disappointment and frustration 27. Lack of recognition 28. Lack of supportive environment 29. Heavy workload, too busy

## Data Availability

Data generated during the present study cannot be shared due to issues of participants’ privacy and confidentiality.
